# Extracorporeal cytokine adsorption: Significant reduction of catecholamine requirement in patients with AKI and septic shock after cardiac surgery

**DOI:** 10.1371/journal.pone.0246299

**Published:** 2021-02-08

**Authors:** Kristina Boss, Michael Jahn, Daniel Wendt, Zaki Haidari, Ender Demircioglu, Matthias Thielmann, Arjang Ruhparwar, Andreas Kribben, Bartosz Tyczynski

**Affiliations:** 1 Department of Nephrology, University Hospital Essen, University Duisburg-Essen, Essen, Germany; 2 Department of Thoracic and Cardiovascular Surgery, West German Heart & Vascular Center, University Hospital Essen, University Duisburg-Essen, Essen, Germany; IRCCS Policlinico S.Donato, ITALY

## Abstract

**Background:**

Extracorporeal cytokine adsorption is an option in septic shock as an additional measure to treat a pathological immune response. Purpose of this study was to investigate the effects of extracorporeal cytokine adsorption on hemodynamic parameters in patients with acute kidney injury (AKI) on continuous renal replacement therapy (CRRT) and septic shock after cardiac surgery.

**Methods:**

In this retrospective study, a total of 98 patients were evaluated. Hemoadsorption was performed by the CytoSorb® adsorber. In all patients cytokine adsorption was applied for at least 15 hours and at least one adsorber was used per patient. To compare cumulative inotrope need in order to maintain a mean arterial pressure (MAP) of ≥ 65 mmHg, we applied vasoactive score (VAS) for each patient before and after cytokine adsorption. A paired t-test has been performed to determine statistical significance.

**Results:**

Before cytokine adsorption the mean VAS was 56.7 points. This was statistically significant decreased after cytokine adsorption (27.7 points, p< 0.0001). Before cytokine adsorption, the mean noradrenalin dose to reach a MAP of ≥ 65 mmHg was 0.49 μg/kg bw/min, the mean adrenalin dose was 0.12 μg/kg bw/min. After cytokine adsorption, significantly reduced catecholamine doses were necessary to maintain a MAP of ≥ 65 mmHg (0.24 μg/kg bw/min noradrenalin; p< 0.0001 and 0.07 μg/kg bw/min adrenalin; p < 0.0001). Moreover, there was a significant reduction of serum lactate levels after treatment (p< 0.0001). The mean SOFA-score for these patients with septic shock and AKI before cytokine adsorption was 16.7 points, the mean APACHE II-score was 30.2 points. The mean predicted in-hospital mortality rate based on this SOFA-score of 16.7 points was 77,0%, respectively 73,0% on APACHE II-score, while the all-cause in-hospital mortality rate of the patients in this study was 59.2%.

**Conclusion:**

In patients with septic shock and AKI undergoing cardiac surgery, extracorporeal cytokine adsorption could significantly lower the need for postoperative inotropes. Additionally, observed versus SOFA- and APACHE II-score predicted in-hospital mortality rate was decreased.

## Introduction

Sepsis and acute kidney injury (AKI) are two major problems in critically ill patients. In nearly 50% of patients presenting AKI, the condition was associated with septic shock. Of the patients with septic shock, up to 60% develop AKI [1]. It is possible that AKI and sepsis present simultaneously at hospital admission or that one entity is evolving during hospitalization. Despite timely and adequate therapy and close monitoring, sepsis-associated acute kidney injury is still associated with significantly higher risk of in-hospital death (odds ratio 1.48) [2].

A central component of the underlying pathomechanism is the release of pro- and anti-inflammatory mediators. This includes e.g. TNF-α, IL1β, IL4, IL6, IL8, as proinflammatory cytokines, where elevated circulating concentrations are linked to increased morbidity and mortality [3]. Since the early 1990s it is known that cytokines can be removed to some extent with conventional continuous renal replacement therapy (CRRT), high volume hemofiltration or high cut-off membranes, but a clinical benefit could not be demonstrated [4–6].

Therefore, we used an extracorporeal cytokine adsorber (CytoSorb®) as an additional measure to treat a pathological immune response in critically ill patients, e.g. presenting endocarditis [7]. In recent years several retrospective studies and case series with a smaller number of cases have been published with different observation variables and endpoints [8–12]. So, the evidence of such a procedure is still limited.

The aim of this retrospective study was to evaluate the effect of extracorporeal cytokine adsorption on hemodynamic parameters in patients with AKI on CRRT and septic shock after cardiac surgery. Therefore, we analysed the noradrenalin and adrenalin demand to maintain a mean arterial pressure (MAP) of ≥ 65 mmHg and serum lactate levels before and after cytokine adsorption. Additionally, we calculated sequential organ failure assessment (SOFA) and acute physiology and chronic health (APACHE II) score before and after cytokine adsorption to assess the therapeutic impact on mortality [13, 14].

## Methods

### Patients

The study was conducted at a cardio-surgical intensive care unit of the West German Heart and Vascular Center, University Hospital Essen, Germany. Observation period was January 2017 to December 2019. All patients included had a severe AKI (KDIGO III) on CRRT and septic shock after cardiac surgery.

In 94% of the patients (92 out of 98) a cardiopulmonary bypass was used. The surgery performed were either a heart valve replacement or reconstruction, bypass surgery, surgery of the thoracic aorta or a combination of these. In six patients implantation of a ventricular assist device, a transapical transcatheter aortic valve implantation (TA-TAVI) or emergency implantation of an extracorporeal membrane oxygenation (ECMO) as a bridging therapy to definitive cardiac surgery were performed. The selection of patients in this study was independent of the cardiac surgery performed.

All patients were initially treated following the Surviving Sepsis Guidelines [15]. If there was no decrease in catecholamine demand after maximum standard therapy, especially including repeated evaluation of septic focus and the possible necessity of septic focus surgery, evaluation and escalation of antibiotic therapy and evaluation of volume status, extracorporeal cytokine adsorption was applied within 24 h of septic shock diagnosis. For extracorporeal cytokine adsorption we used the CytoSorb® adsorber (Cytosorbents®, Monmouth Junction, NJ, USA). CRRT and installation of the adsorber in line to the CRRT circuit were performed according to standard care and according to the standard procedures in our hospital. Blood flow rate was set at 100 ml/h, the CRRT was performed as continuous veno-venous hemodialysis. The anticoagulation was mostly citrate-based.

For analysis we defined two different periods of time: 24 hours before the first and 24 hours after the last cytokine adsorption. 22 out of 98 patients died before 24 hours had elapsed after the last cytokine adsorption. Therefore, comparisons between the two time periods with regard to catecholamine requirement and serum lactate level included only 76 patients who survived longer than 24 hours after the last cytokine adsorption. Prognostic scores were applied for all 98 patients enrolled in the study. Comparisons of the prognostic scores between the two periods of time include the 76 patients who survived longer than 24 hours after the last cytokine adsorption.

### Definition

Septic shock was defined as sepsis with persisting hypotension requiring vasopressors to maintain MAP of ≥ 65 mmHg and having a serum lactate level of ≥ 2 mmol/L (18 mg/dL) despite adequate volume resuscitation [15]. Acute kidney injury was defined analogous to the KDIGO criteria using serum creatinine and urine output or need of renal replacement therapy (RRT) [16].

### Statistical analysis

The data were analysed retrospectively in anonymous form. Data are expressed as mean value ± standard error of the mean (SEM), if not declared otherwise. The D´Agostino-Pearson Test excluded normality of some data sets. In these cases, the Wilcoxon matched-pairs signed rank test was used for comparison of means. Otherwise a paired-t test was used. Statistical significance was determined with a p-value < 0.05. For statistical analysis and graphical evaluation, the software GraphPad Prism® 8 was used.

### Ethics approval

The study was performed in accordance with the Declaration of Helsinki and the International Conference on Harmonization Good Clinical Practice guidelines. The study was approved by the local ethics committee of the University of Duisburg-Essen (18-8497-BO).

## Results

### Patient characteristics

**Fig 1:**
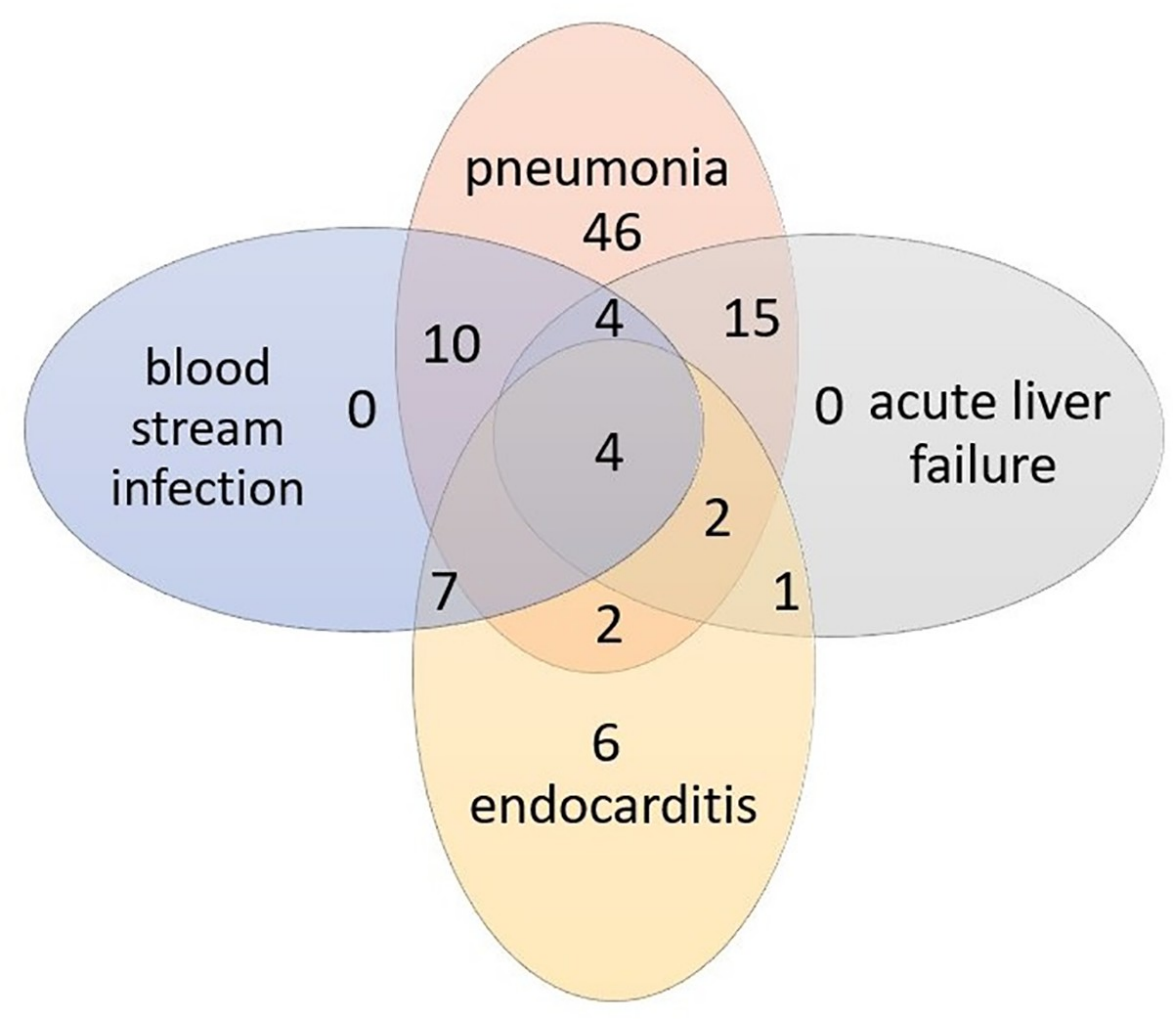
Underlying medical diseases of the patients. Figure shows number of patients depending of the underlying medical diseases.

A total of 98 patients were evaluated in this study. 60% of the patients were male (59 out of 98), 40% female (39 out of 98). The average age was 67 years (range 33–83 years). Detailed patient characteristics can be found in Table 1. The number of patients with their underlying medical diseases is shown in Fig 1. The most common sepsis- entity in the study population was pneumosepsis with 84 out of 98 patients (86%).

**Table 1:** Patients characteristics.

Characteristics	Patients (n = 98)
Demography	
Age [years], mean (range)	67, 33–83
Male, n (%)	59 (60%)
Female, n (%)	39 (40%)
Comorbidity, n (%)	
No CKD	54
CKD G2	4
CKD G3	13
CKD G4	16
CKD G5D	11
Diabetes requiring insulin	33
Other severe lung disease	11
Severe pulmonal hypertension of ≥ 40 mmHg systolic	4
Liver cirrhosis	8
Heart failure NYHA IV	5
Portal hypertension	2
Previous hepatic encephalopathy	1
Cardiac surgery	
Emergency surgery, n	44
Elective surgery, n	54
Cardiopulmonary bypass, n (time [mean ± SD])	92 (194 ± 112 min)
Heart valve surgery, n	63
Surgery on thoracic aorta, n	25
Bypass operation	55
Underlying medical disease, n (%)	
Pneumonia	83
Blood stream infection	25
Acute liver failure	26
Endocarditis	22
Other types of infection (eg. Catheter-associated infection, genitourinary infection)	8
Days on ICU, mean (range)	16 d (1–142 d)
Days on ICU before cytokine adsorption, mean	4 d

To characterize patient’s mortality risk due to the cardiac surgery performed, we applied EuroSCORE II and formed three different risk groups; EuroSCORE II ≤ 4% (low risk), 4–9% (intermediate risk) and of ≥ 9% (high risk) [17]. The mean EuroSCORE II was 19.3% (range 0.62 to 83.97%). 51% of the patients had a high mortality risk over 9% (Table 2).

**Table 2:** EuroSCORE II risk groups.

EuroSCORE II risk group	n (%)	mean % (range)
low risk (< 4%)	30 (30.6%)	2.4% (0.62–3.82)
Intermediate risk (4–9%)	18 (18.4%)	7.34% (4.11–8.96)
high risk (of ≥ 9%)	50 (51.0%)	33.7% (9.14–83.97)

Cytokine adsorption was applied over 15–40 hours with one to four adsorbers used per patient. All patients underwent additional treatment depending on their clinical response. Adsorbers were mainly changed every 24 hours. Application time of less than 24 hours was due to the necessity of surgical revision, radiology examination or death.

### Cytokine adsorption: Catecholamine requirement and blood lactate levels

To compare cumulative catecholamine dose, we applied vasoactive score (VAS) for each patient for both periods of time. This score quantifies the amount of medical cardiovascular support required by a patient and includes dopamine, dobutamine, adrenalin, milrinone, vasopressin and noradrenalin. It is established in patients after cardiac surgery as well as in sepsis patients [18–20].

Of the initial 98 patients, 22 died before 24 hours after the last cytokine adsorption had passed. Thus, the catecholamine requirement of 76 patients before and after cytokine adsorption could be evaluated. From these 76 survivors, 56 received adrenalin, 71 received noradrenalin and 5 received dobutamin before cytokine adsorption.

**Fig 2:**
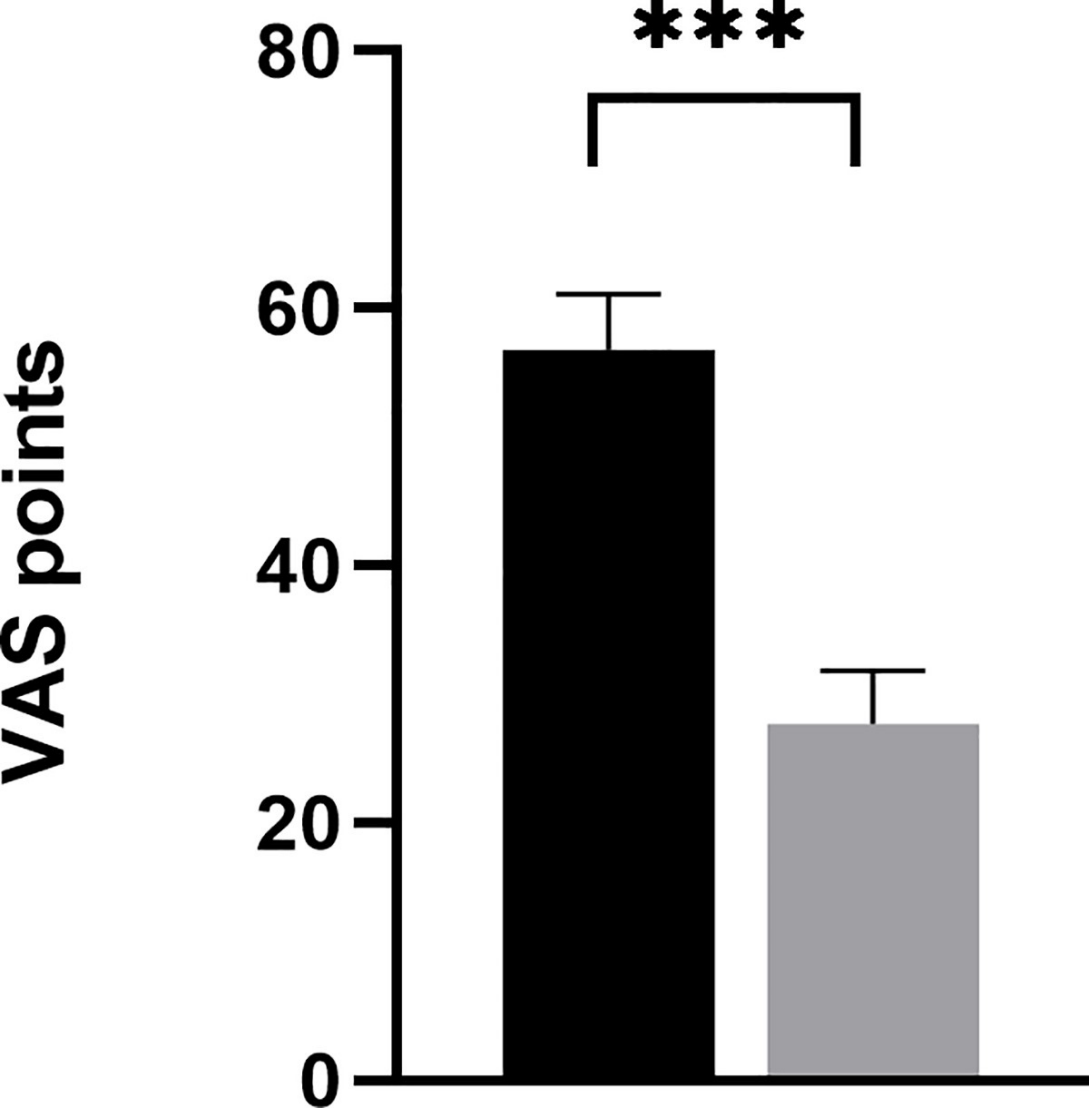
Vasoactive score (VAS) for catecholamines before and after cytokine adsorption. Graph shows vasoactive score (VAS) for catecholamines required to reach a mean arterial pressure (MAP) ≥ 65 mmHg before and after cytokine adsorption. Data include n = 76 patients. Error bars correspond to SEM. Asterisks mark differences between both groups at a significance ***p < 0.01.

The mean VAS before cytokine adsorption was 56.7 points. The mean VAS after cytokine adsorption was 27.7 points. Therefore, the group of survivors showed a VAS reduction of 51.1%, which means a highly statistically significant decrease with p < 0.001 (Fig 2).

**Fig 3:**
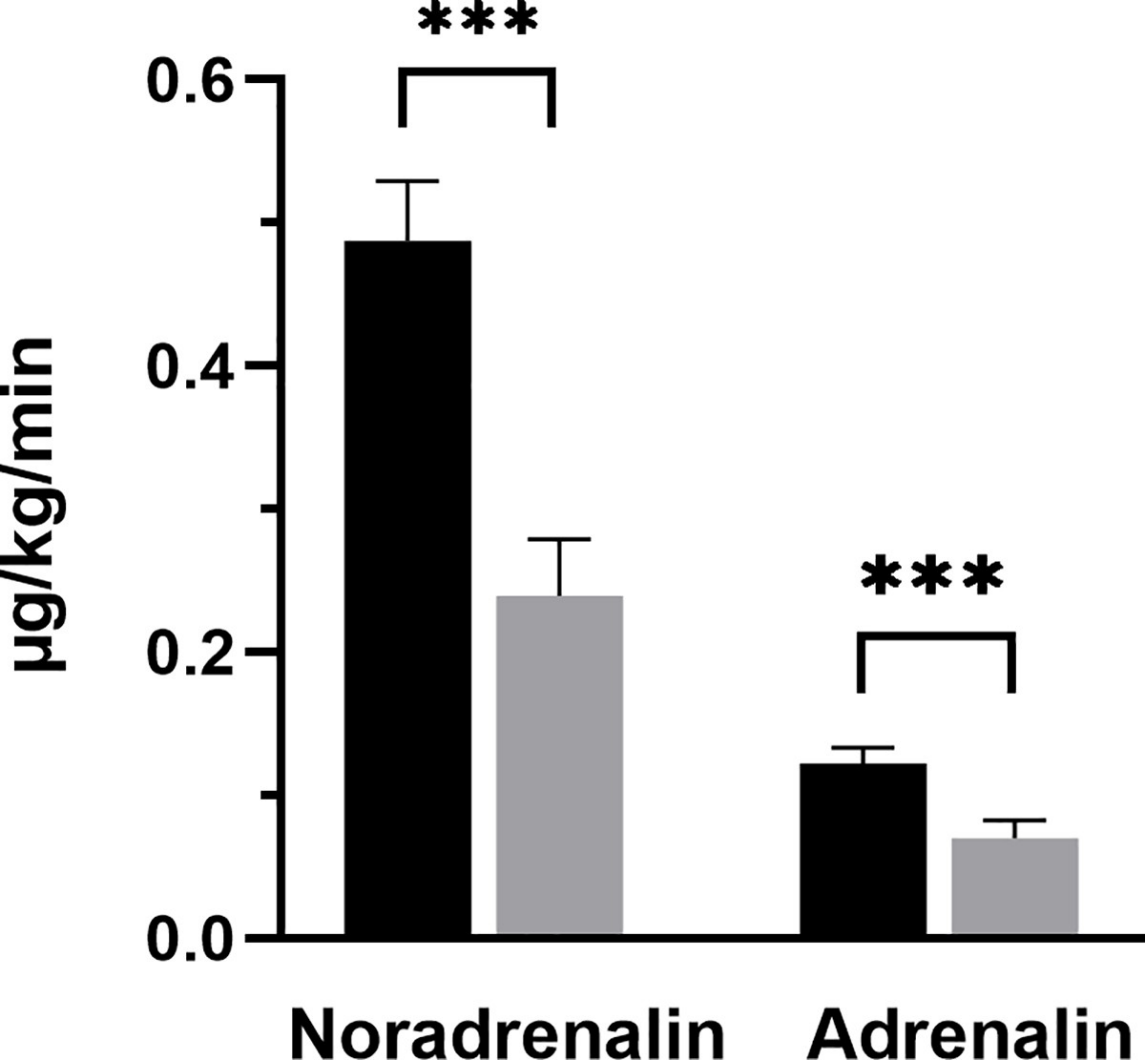
Noradrenalin and adrenalin requirement before and after cytokine adsorption. Graphs show mean noradrenalin and adrenalin requirement before and after cytokine adsorption. Data shows n = 71 patients (noradrenalin) and n = 56 patients (adrenalin). Error bars correspond to SEM. Asterisks mark differences between both groups at a significance ***p < 0.001.

Before cytokine adsorption 71 out of 76 patients needed noradrenalin to reach a MAP of ≥ 65 mmHg. The mean noradrenalin dose was 0.49 μg/kg bw/min. After cytokine adsorption, 61 patients needed noradrenalin to maintain a MAP of ≥ 65 mmHg. The mean noradrenalin dose after cytokine adsorption was 0.24 μg/kg bw/min, which shows a highly statistical significantly decrease (p< 0.0001) (Fig 3).

Before cytokine adsorption 56 out of 76 patients needed adrenalin to reach a MAP of ≥ 65 mmHg. The mean adrenalin dose was 0.12 μg/kg bw/min. After cytokine adsorption, only 44 patients needed adrenalin to maintain a MAP of ≥ 65 mmHg. The mean adrenalin dose after cytokine adsorption was 0.07 μg/kg bw/min, which corresponds also to a statistical significantly decrease (p < 0.0001) (Fig 3).

In order to evaluate which group of patients had the most benefit on hemodynamic parameters, we related relative VAS reduction to the prognostic scores performed. The highest relative VAS-reduction could be observed in patients with lowest EuroSCORE II and SOFA- and APACHE II-score. But also, in high-risk patients a reduction of VAS after cytokine adsorption of about 70% could be achieved (Table 3).

**Table 3:** Relative vasoactive score (VAS) reduction.

Score	Risk group	n	Relative VAS reduction [%]
**EuroSCORE II**	low risk (< 4%)	21	72.15
	Intermediate risk (4–9%)	14	67.75
	high risk (of ≥ 9%)	27	67.0
**APACHE II**	18 points	1	100
	21–24 points	5	61,84
	25–29 points	21	64.17
	30–34 points	29	66.66
	of ≥ 34 points	6	73.22
**SOFA**	13–14 points	10	82.97
	15–16 points	18	66.53
	17 points	13	58.85
	18–22 points	21	71.15

62 out of 76 patients analysed showed a reduced catecholamine requirement after cytokine adsorption. In 14 patients the catecholamine demand was increased. In addition to AKI and sepsis, these patients suffered an additional hit with, for example, acute liver failure or a multi-infarction syndrome. In these cases, cytokine adsorption was used as a last therapeutic option for severely ill patients with very poor prognosis.

Beyond that, in the study cohort there is a subgroup of patients, who developed septic shock despite elective surgery and low risk EuroSCORE II. The reasons for this are very individually but long cardiopulmonary bypass times, the need for IABP or ECMO or overall long surgery times for complex cardio-surgical procedures were often founded. Also, there were some patients, who developed a low cardiac output syndrome after surgery and/or needed prolonged ventilation.

**Fig 4:**
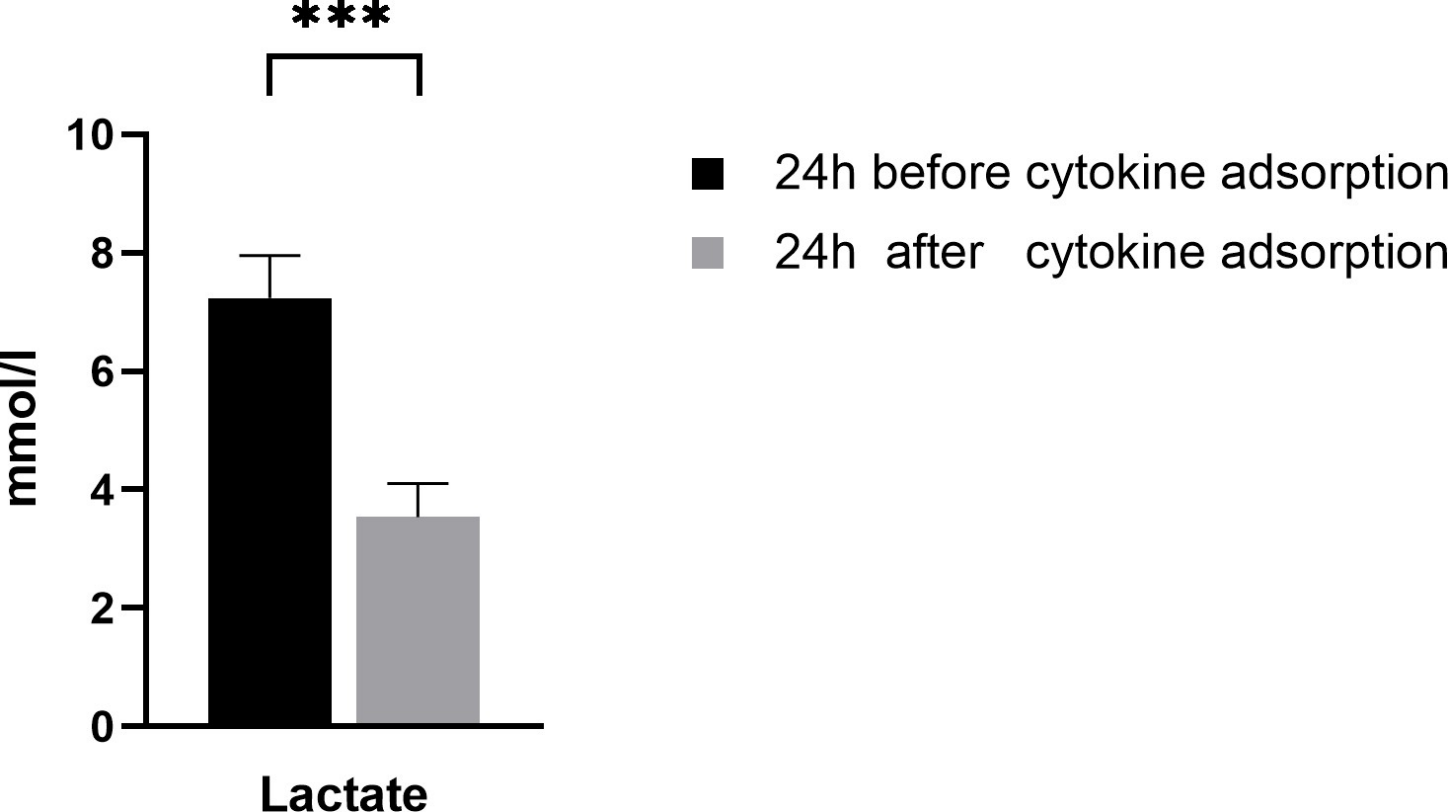
Serum lactate levels before and after cytokine adsorption. Graphs show mean serum lactate level before and after cytokine adsorption. Data shows n = 76 patients. Error bars correspond to SEM. Asterisks mark differences between both groups at a significance ***p < 0.001.

The mean serum lactate level before cytokine adsorption was 6.7 mmol/l, after treatment 3.6 mmol/l, which corresponds to a statistically significant reduction (p < 0.0001) (Fig 4, Table 4).

**Table 4:** Hemodynamic parameters.

	24 h before cytokine adsorption (mean)	24 h after cytokine adsorption (mean)
**Noradrenalin**	0.49 μg/kg bw/min	0.24 μg/kg bw/min
**Adrenalin**	0.12 μg/kg bw/min	0.07 μg/kg bw/min
**Serum lactate**	7.2 mmol/l	3.5 mmol/l

### Cytokine adsorption: Impact on mortality

In this study we investigated the most severe cases of patients with septic shock and AKI on CRRT after cardiac surgery, which had a very high score-predicted mortality. Of the 98 patients examined in this study, 22 died before 24 hours had elapsed after the last cytokine adsorption. Another 39 patients died within hospital stay. According to current guidelines for reporting mortality after valve interventions, all of the 61 patients died, died within 30 days after cardiac surgery [21]. No device-related adverse events were observed.

**Fig 5:**
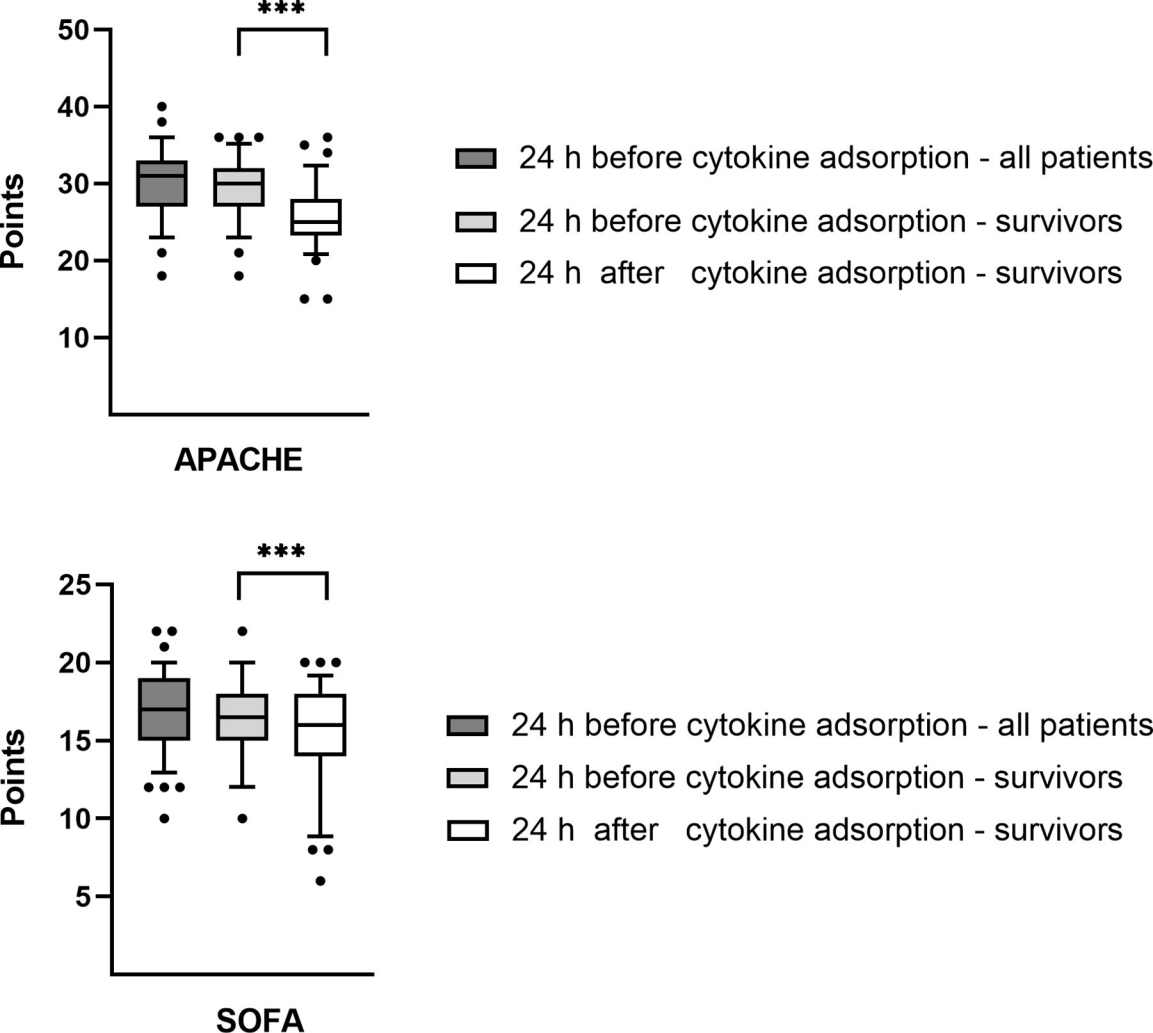
SOFA- score (Fig 5A) and APACHE II-score (Fig 5B) before and after cytokine adsorption. First box shows all n = 98 patients analysed in the study. Second and third box show n = 76 patients who survived longer than 24 hours after cytokine adsorption. Central lines denote median values, and upper and lower borders represent 25th and 75th percentiles. The whiskers represent 5–95 percentile. Highest and lowest values are marked as dots. Asterisks mark differences between both groups at a significance ***p < 0.001.

The mean SOFA-score before cytokine adsorption of all 98 patients with septic shock and AKI evaluated in this study was 16.7 points. The mean predicted in-hospital mortality rate based on this SOFA-score of 16.7 points was 77.0% [22] while the all-cause in-hospital mortality rate of the patients in this study was 59.2%. The mean SOFA-score of the 76 patients who survived longer than 24 hours after cytokine adsorption was 16.3 points before, respectively 15.2 points after cytokine adsorption (Fig 5).

In addition, we applied the APACHE II-score. Before cytokine adsorption the mean APACHE II-score for all 98 evaluated patients was 30.2 points, which corresponds to an in-hospital mortality rate of about 73.0% [13]. Thus, the APACHE II-score predicted mortality rate was also higher than the observed mortality rate. The mean APACHE II-score of the 76 patients who survived longer than 24 hours after cytokine adsorption was 29.4 points before, respectively 25.6 points after cytokine adsorption (Fig 5, Table 5).

**Table 5:** Prognostic scores.

	24 h before cytokine adsorption–all patients mean (range)	24 h before cytokine adsorption–Survivors mean (range)	24 h after cytokine adsorption–Survivors mean (range)
**SOFA**	16.7 (10–22)	16.3 (10–22)	15.2 (6–20)
**APACHE II**	30.2 (18–40)	29.4 (18–36)	25.6 (15–36)

Thus, there was a significantly reduction in mean SOFA- and APACHE II-score after cytokine adsorption (SOFA-score p < 0.001, APACHE II-score p < 0.001).

## Discussion

### Key findings

In this study we evaluated the effects of extracorporeal cytokine adsorption on hemodynamic parameters in patients with AKI on CRRT and septic shock after cardiac surgery. Extracorporeal cytokine adsorption could significantly lower the need for postoperative inotropes. Also, we found a significant reduction of serum lactate level after treatment. In addition, mean SOFA- and APACHE II-score were significantly decreased after cytokine adsorption and the observed in-hospital mortality rate was lower than SOFA- and APACHE II-score predicted mortality rate. In these patients with a predicted very high mortality risk according to EuroSCORE II, SOFA- and APACHE II-score, extracorporeal cytokine adsorption stabilized hemodynamic parameters with an average reduction of VAS by 27.7 points.

### Comparison with previous studies

There are several studies, which evaluated the use of CytoSorb® adsorber in the setting of sepsis. First Schädler et al. showed a significant IL-6 reduction as a proinflammatory cytokine, however, the results were rather disadvantageous in terms of mortality [8]. In 2017 Friesecke et al. could show, that a significant reduction of catecholamine requirement can be achieved by using only one adsorber [10]. This was confirmed in a smaller group of sepsis patients [11] and underlines the results of the present study. Thus, a stabilization of hemodynamic parameters in sepsis patients by reduction of catecholamine requirement by cytokine adsorption seems possible in a majority of cases. Apart from a reduced need for catecholamine, the effect on mortality was investigated in different ways. Kogelmann et al. applied the APACHE II-score and showed a lower observed than APACHE II-score predicted mortality [11]. This was confirmed in a randomized controlled trial, in which the observed mortality rate was significantly lower than the mortality rate predicted by SOFA- score [12]. In the present study, both SOFA- and APACHE II-score were applied and evaluated before and after cytokine adsorption. Our results confirm the results from former studies; observed versus score-predicted mortality rate is decreased and additionally, mean SOFA- and APACHE II-score is significantly lower after cytokine adsorption.

In addition, cytokine adsorption by CytoSorb ® adsorber was also investigated in patients after cardiac surgery. However, previous studies in this setting have shown inconsistent results. A randomized controlled trial with 37 patients undergoing elective cardiac surgery showed no differences in IL-6 level, catecholamine requirement or mortality [23]. On the other hand, several retrospective case series demonstrated a stabilization of hemodynamic parameters by reduction of vasopressors and cytokine levels [24–26]. In these studies, comparable patients as in the present study were examined. Therefore, this study can confirm the results obtained so far.

### Strength and limitations

The present study has several strengths and limitations. To our knowledge, this is the largest patient population with AKI and sepsis after cardiac surgery examined, compared to previous studies. For the first time, an analysis was carried out to determine which subgroups benefit to what extent from cytokine adsorption to stabilize hemodynamic parameters. The results are limited by the monocentric retrospective study design and need to be confirmed in controlled studies.

## Conclusion

The present study could show a stabilization of hemodynamic parameters in patients with AKI on CRRT and sepsis after cardiac surgery with a significant reduction of catecholamine requirement and serum lactate level. This was also observed in patients with very high score-predicted mortality. Additionally, observed versus SOFA- and APACHE II-score predicted in-hospital mortality rate was decreased.

## Supporting information

S1 File (DOCX)

## Abbreviations

AKI: Acute kidney injury
APACHE II: Acute physiology and chronic health
CKD: Chronic kidney disease
CRRT: Continuous renal replacement therapy
ECMO: Extracorporeal membrane oxygenation
ICU: Intensive care unit
IL: Interleukin
MAP: Mean arterial pressure
NYHA: New York Heart Association
SEM: Standard error of the mean
SOFA: Sequential organ failure assessment
TA-TAVI: Transapical transcatheter aortic valve implantation
TNF: Tumor necrosis factor
VAS: Vasoactive score
