# Supplementary material for: Extracorporeal cytokine adsorption: Significant reduction of catecholamine requirement in patients with AKI and septic shock after cardiac surgery
Source: PLoS One. 2021 Feb 8;16(2):e0246299. doi: 10.1371/journal.pone.0246299 (PMC7870055; doi:10.1371/journal.pone.0246299)
Supplement: S1 File — (DOCX) [file pone.0246299.s001.docx]

| Noradrenalin pre | Noradrenalin post |  | Adrenalin pre | Adrenalin post |  |  |  |  |  |
| --- | --- | --- | --- | --- | --- | --- | --- | --- | --- |
| 0,6 | 0 |  |  |  |  |  |  |  |  |
| 0,7 | 0,307 |  | 0,052 | 0,052 |  |  |  |  |  |
| 0,9 | 0,05 |  | 0,05 | 0,18 |  |  |  |  |  |
| 1,2 | 0,14 |  | 0,15 | 0,1 |  |  |  |  |  |
| 0,75 | 0,25 |  |  |  |  |  |  |  |  |
| 0,35 | 0 |  | 0,06 | 0,12 |  |  |  |  |  |
| 0,4 | 0,07 |  | 0,05 | 0,02 |  |  |  |  |  |
| 1,16 | 1,5 |  |  |  |  |  |  |  |  |
| 0,2 | 0,3 |  | 0 | 0,08 |  |  |  |  |  |
| 0,6 | 0,35 |  | 0,15 | 0,09 |  |  |  |  |  |
| 0 | 0,05 |  |  |  |  |  |  |  |  |
| 0,8 | 0,14 |  | 0,2 | 0,15 |  |  |  |  |  |
| 0,1 | 1,45 |  | 0,1 | 0,1 |  |  |  |  |  |
| 0 | 1,4 |  | 0 | 0,6 |  |  |  |  |  |
| 0,4 | 0 |  | 0,08 | 0 |  |  |  |  |  |
| 0,5 | 0,14 |  | 0,2 | 0,08 |  |  |  |  |  |
| 0,5 | 0 |  |  |  |  |  |  |  |  |
| 0,1 | 0,14 |  | 0,3 | 0 |  |  |  |  |  |
| 0,75 | 0,05 |  | 0,08 | 0,08 |  |  |  |  |  |
| 0,23 | 0,06 |  | 0,2 | 0,06 |  |  |  |  |  |
| 0,5 | 0,02 |  | 0,08 | 0 |  |  |  |  |  |
| 0,4 | 0,05 |  | 0,15 | 0 |  |  |  |  |  |
| 0,5 | 0,43 |  | 0,02 | 0,05 |  |  |  |  |  |
| 0,3 | 0,01 |  | 0,15 | 0,08 |  |  |  |  |  |
| 0,49 | 0,04 |  | 0,2 | 0,04 |  |  |  |  |  |
| 1 | 0,6 |  |  |  |  |  |  |  |  |
| 1,2 | 0,3 |  | 0,12 | 0,1 |  |  |  |  |  |
| 0,35 | 0,33 |  | 0,2 | 0,1 |  |  |  |  |  |
| 0,15 | 0 |  | 0,03 | 0,02 |  |  |  |  |  |
| 0,29 | 0,7 |  | 0,18 | 0,2 |  |  |  |  |  |
| 0,3 | 0,04 |  | 0,1 | 0,01 |  |  |  |  |  |
| 0,5 | 0,5 |  | 0,2 | 0,2 |  |  |  |  |  |
| 0 | 0,02 |  |  |  |  |  |  |  |  |
| 0,37 | 0,38 |  | 0,3 | 0,2 |  |  |  |  |  |
| 0,18 | 0,8 |  | 0,07 | 0,1 |  |  |  |  |  |
| 0,01 | 0 |  |  |  |  |  |  |  |  |
| 0,6 | 0,16 |  | 0,05 | 0,02 |  |  |  |  |  |
| 0,01 | 0,08 |  | 0,01 | 0,06 |  |  |  |  |  |
| 0,1 | 0,18 |  | 0,05 | 0 |  |  |  |  |  |
| 0,45 | 0,01 |  | 0,03 | 0,03 |  |  |  |  |  |
| 0,8 | 0 |  | 0,1 | 0,05 |  |  |  |  |  |
| 0,2 | 0,02 |  | 0,05 | 0,02 |  |  |  |  |  |
| 0,3 | 0,09 |  | 0,15 | 0 |  |  |  |  |  |
|  |  |  |  |  |  |  |  |  |  |
| 1,1 | 0,15 |  | 0,03 | 0 |  |  |  |  |  |
| 0,35 | 0,27 |  |  |  |  |  |  |  |  |
| 0,2 | 0,03 |  | 0,17 | 0 |  |  |  |  |  |
| 0,13 | 0,05 |  | 0,4 | 0,02 |  |  |  |  |  |
|  |  |  |  |  |  |  |  |  |  |
| 0,5 | 0 |  | 0,3 | 0 |  |  |  |  |  |
| 0,5 | 0,27 |  | 0,2 | 0,08 |  |  |  |  |  |
| 0,28 | 0,15 |  | 0,1 | 0,1 |  |  |  |  |  |
| 0,29 | 0,24 |  | 0,17 | 0,07 |  |  |  |  |  |
| 0,4 | 0,15 |  | 0,1 | 0 |  |  |  |  |  |
| 0,2 | 0 |  |  |  |  |  |  |  |  |
| 0,81 | 0,18 |  | 0,2 | 0,1 |  |  |  |  |  |
| 1 | 0,36 |  | 0,05 | 0,09 |  |  |  |  |  |
| 2 | 0,07 |  | 0,16 | 0,05 |  |  |  |  |  |
| 0,34 | 0,08 |  | 0,06 | 0,05 |  |  |  |  |  |
| 0,82 | 0,37 |  |  |  |  |  |  |  |  |
| 0,3 | 0,08 |  |  |  |  |  |  |  |  |
| 0,2 | 0,05 |  | 0,1 | 0,18 |  |  |  |  |  |
| 0,42 | 1 |  | 0,08 | 0,01 |  |  |  |  |  |
| 0,6 | 0,07 |  | 0,1 | 0 |  |  |  |  |  |
| 0,35 | 0 |  | 0,1 | 0 |  |  |  |  |  |
| 0,85 | 0,65 |  | 0,12 | 0 |  |  |  |  |  |
| 0,25 | 0 |  | 0,18 | 0,04 |  |  |  |  |  |
| 0,23 | 0 |  | 0,25 | 0,2 |  |  |  |  |  |
| 0,8 | 0,6 |  | 0,1 | 0,02 |  |  |  |  |  |
| 0,8 | 0,02 |  | 0,02 | 0,02 |  |  |  |  |  |
| 0,6 | 0,18 |  | 0,08 | 0,03 |  |  |  |  |  |
| 1 | 0,15 |  | 0,1 | 0 |  |  |  |  |  |
| 0,6 | 1 |  |  |  |  |  |  |  |  |
| 0,75 | 0,27 |  |  |  |  |  |  |  |  |
| 0,1 | 0,1 |  |  |  |  |  |  |  |  |
| 0,05 | 0 |  |  |  |  |  |  |  |  |

| VAS pre | VAS post |
| --- | --- |
| 60 | 0 |
| 75,2 | 35,9 |
| 95 | 23 |
| 135 | 24 |
| 75 | 25 |
| 41 | 12 |
| 45 | 9 |
| 116 | 15 |
| 20 | 38 |
| 75 | 44 |
| 0 | 5 |
| 100 | 29 |
| 20 | 155 |
| 0 | 200 |
| 48 | 0 |
| 70 | 22 |
| 50 | 0 |
| 40 | 14 |
| 83 | 13 |
| 43 | 12 |
| 58 | 2 |
| 55 | 5 |
| 52 | 48 |
| 45 | 9 |
| 69 | 8 |
| 100 | 60 |
| 132 | 40 |
| 55 | 43 |
| 18 | 2 |
| 47 | 90 |
| 40 | 5 |
| 70 | 70 |
| 0 | 2 |
| 67 | 58 |
| 25 | 90 |
| 1 | 0 |
| 65 | 18 |
| 2 | 14 |
| 15 | 18 |
| 48 | 4 |
| 90 | 5 |
| 25 | 4 |
| 45 | 9 |
| 0 | 0 |
| 113 | 15 |
| 35 | 27 |
| 37 | 3 |
| 53 | 7 |
| 0 | 0 |
| 80 | 0 |
| 70 | 35 |
| 38 | 25 |
| 46 | 31 |
| 50 | 15 |
| 20 | 0 |
| 101 | 28 |
| 105 | 45 |
| 50 | 13 |
| 88 | 42 |
| 30 | 8 |
| 20 | 5 |
| 52 | 118 |
| 68 | 8 |
| 45 | 0 |
| 95 | 65 |
| 0 | 37 |
| 41 | 4 |
| 105 | 80 |
| 90 | 4 |
| 62 | 20 |
| 18 | 13 |
| 15 | 0 |
| 203,5 | 10,5 |
| 109 | 23 |
| 74 | 109 |
| 84 | 33 |

| APACHE all | APACHE survivor pre | APACHE survivor post |
| --- | --- | --- |
| 34 | 25 | 21 |
| 33 | 35 | 31 |
| 25 | 35 | 27 |
| 35 | 32 | 25 |
| 35 | 30 | 28 |
| 32 | 31 | 27 |
| 30 | 31 | 25 |
| 32 | 28 | 22 |
| 31 | 32 | 25 |
| 40 | 28 | 26 |
| 31 | 36 | 30 |
| 28 | 31 | 26 |
| 30 | 32 | 29 |
| 32 | 30 | 25 |
| 28 | 28 | 23 |
| 36 | 29 | 23 |
| 31 | 18 | 20 |
| 34 | 21 | 15 |
| 32 | 26 | 22 |
| 32 | 36 | 28 |
| 30 | 34 | 26 |
| 28 | 25 | 23 |
| 29 | 33 | 28 |
| 18 | 27 | 26 |
| 21 | 31 | 25 |
| 31 | 34 | 25 |
| 26 | 31 | 25 |
| 36 | 32 | 28 |
| 34 | 27 | 27 |
| 25 | 30 | 24 |
| 33 | 25 | 24 |
| 27 | 24 | 23 |
| 31 | 23 | 22 |
| 34 | 27 | 22 |
| 31 | 30 | 25 |
| 27 | 25 | 24 |
| 32 | 24 | 23 |
| 32 | 27 | 26 |
| 27 | 30 | 28 |
| 31 | 26 | 25 |
| 30 | 30 | 28 |
| 25 | 35 | 24 |
| 29 | 23 | 24 |
| 24 | 30 | 24 |
| 23 | 29 | 26 |
| 27 | 31 | 25 |
| 30 | 23 | 22 |
| 25 | 28 | 21 |
| 38 | 29 | 23 |
| 24 | 36 | 26 |
| 27 | 31 | 24 |
| 30 | 33 | 35 |
| 26 | 27 | 31 |
| 30 | 35 | 28 |
| 35 | 30 | 24 |
| 23 | 28 | 34 |
| 30 | 31 | 32 |
| 29 | 31 | 27 |
| 31 | 34 | 25 |
| 23 | 30 | 32 |
| 28 | 34 | 31 |
| 29 | 31 | 24 |
| 36 | 28 | 28 |
| 31 | 28 | 25 |
| 33 | 33 | 22 |
| 27 | 32 | 36 |
| 31 | 25 | 24 |
| 35 | 31 | 27 |
| 30 | 32 | 29 |
| 33 | 34 | 27 |
| 28 | 27 | 25 |
| 31 | 34 | 31 |
| 31 | 25 | 23 |
| 34 | 26 | 23 |
| 30 | 26 | 25 |
| 34 | 25 | 15 |
| 31 |  |  |
| 28 |  |  |
| 28 |  |  |
| 33 |  |  |
| 34 |  |  |
| 32 |  |  |
| 25 |  |  |
| 31 |  |  |
| 36 |  |  |
| 32 |  |  |
| 34 |  |  |
| 27 |  |  |
| 34 |  |  |
| 25 |  |  |
| 26 |  |  |
| 36 |  |  |
| 34 |  |  |
| 32 |  |  |
| 32 |  |  |
| 26 |  |  |
| 34 |  |  |
| 25 |  |  |

| SOFA all | SOFA survivors pre | SOFA survivors post |
| --- | --- | --- |
| 14 | 15 | 9 |
| 13 | 16 | 15 |
| 15 | 16 | 16 |
| 16 | 17 | 17 |
| 16 | 17 | 16 |
| 17 | 22 | 20 |
| 17 | 19 | 17 |
| 19 | 19 | 18 |
| 22 | 14 | 19 |
| 16 | 16 | 19 |
| 19 | 12 | 11 |
| 19 | 19 | 16 |
| 18 | 20 | 18 |
| 14 | 12 | 15 |
| 16 | 16 | 13 |
| 12 | 17 | 16 |
| 19 | 14 | 14 |
| 19 | 16 | 14 |
| 20 | 15 | 17 |
| 19 | 19 | 17 |
| 12 | 18 | 18 |
| 16 | 14 | 14 |
| 17 | 17 | 16 |
| 14 | 15 | 15 |
| 16 | 17 | 16 |
| 21 | 18 | 17 |
| 15 | 20 | 18 |
| 19 | 17 | 17 |
| 18 | 16 | 13 |
| 14 | 17 | 13 |
| 17 | 19 | 17 |
| 15 | 20 | 18 |
| 17 | 12 | 15 |
| 18 | 17 | 17 |
| 20 | 17 | 19 |
| 19 | 15 | 6 |
| 16 | 16 | 19 |
| 17 | 17 | 14 |
| 16 | 15 | 16 |
| 18 | 14 | 14 |
| 17 | 17 | 18 |
| 19 | 13 | 11 |
| 19 | 19 | 20 |
| 20 | 13 | 13 |
| 12 | 14 | 14 |
| 17 | 15 | 14 |
| 17 | 15 | 13 |
| 15 | 19 | 15 |
| 22 | 10 | 9 |
| 16 | 14 | 9 |
| 17 | 17 | 16 |
| 15 | 13 | 16 |
| 14 | 15 | 18 |
| 17 | 16 | 16 |
| 13 | 17 | 11 |
| 19 | 16 | 18 |
| 13 | 19 | 19 |
| 14 | 19 | 19 |
| 15 | 18 | 14 |
| 15 | 17 | 18 |
| 19 | 18 | 18 |
| 10 | 19 | 16 |
| 14 | 16 | 16 |
| 17 | 16 | 14 |
| 13 | 13 | 8 |
| 15 | 17 | 20 |
| 19 | 15 | 9 |
| 16 | 18 | 16 |
| 17 | 19 | 18 |
| 18 | 19 | 15 |
| 16 | 17 | 14 |
| 19 | 18 | 17 |
| 19 | 15 | 13 |
| 18 | 14 | 12 |
| 17 | 15 | 14 |
| 18 | 14 | 8 |
| 19 |  |  |
| 16 |  |  |
| 16 |  |  |
| 13 |  |  |
| 18 |  |  |
| 17 |  |  |
| 15 |  |  |
| 18 |  |  |
| 19 |  |  |
| 19 |  |  |
| 19 |  |  |
| 17 |  |  |
| 18 |  |  |
| 15 |  |  |
| 14 |  |  |
| 19 |  |  |
| 18 |  |  |
| 17 |  |  |
| 17 |  |  |
| 15 |  |  |
| 18 |  |  |
| 14 |  |  |

| Lactate pre | Lactate post |
| --- | --- |
| 25 | 0,7 |
| 3,8 | 0,5 |
| 3,5 | 2,6 |
| 3,8 | 2 |
| 9,3 | 2,5 |
| 7,9 | 6 |
| 2,5 | 4 |
| 4,5 | 6 |
| 3,4 | 3,9 |
| 2,1 | 4,4 |
| 7 | 1,7 |
| 5 | 12,5 |
| 1 | 29 |
| 8 | 4,3 |
| 7,5 | 2,8 |
| 3,6 | 2,7 |
| 3,9 | 1,5 |
| 7 | 26 |
| 13,6 | 3,9 |
| 4,1 | 2,1 |
| 4,4 | 1,4 |
| 11,8 | 1,3 |
| 5 | 2,8 |
| 10,4 | 2 |
| 5,1 | 1,9 |
| 3,5 | 2,5 |
| 28 | 7,6 |
| 11,6 | 1,8 |
| 24 | 2,2 |
| 6 | 2,1 |
| 2,1 | 0,9 |
| 4,3 | 2,1 |
| 1 | 1,2 |
| 10,9 | 2,6 |
| 4,5 | 1,5 |
| 1,3 | 0,5 |
| 5,3 | 3,2 |
| 4,9 | 1,2 |
| 2,2 | 2,2 |
| 2,2 | 2 |
| 9,6 | 1,9 |
| 2 | 1 |
| 10,6 | 2,4 |
| 1,7 | 1,3 |
| 4,8 | 1,2 |
| 5 | 1,4 |
| 6,5 | 1 |
| 5,8 | 1,7 |
| 2 | 1,7 |
| 11,7 | 2,7 |
| 8,8 | 3,2 |
| 30 | 19 |
| 10,8 | 3,5 |
| 8,8 | 0,9 |
| 1,3 | 0,8 |
| 7 | 3,8 |
| 1 | 2,2 |
| 4,8 | 1,5 |
| 12 | 2,4 |
| 14,7 | 2,1 |
| 10,9 | 5 |
| 1,9 | 1,8 |
| 5,4 | 2 |
| 2,7 | 1,4 |
| 5,2 | 0,8 |
| 19 | 4 |
| 2,4 | 1,7 |
| 6,7 | 2,5 |
| 9,9 | 3,1 |
| 2,7 | 3 |
| 1,9 | 1 |
| 1,5 | 2,4 |
| 11,2 | 2,5 |
| 2 | 1,1 |
| 14 | 16 |
| 19 | 2,5 |
